# Delayed elective nodal irradiation in head-and-neck cancer radiotherapy reduces acute lymphocyte damage, lymphopenia, and modulates immune signatures

**DOI:** 10.1016/j.ctro.2026.101315

**Published:** 2026-10-08

**Authors:** Justus Kaufmann, Ahmed Salah, Federico Marini, Sophia Drabke, Nina Gercek, Stephanie Breinich, Laura Oebel, Heinz Schmidberger, Sebastian Zahnreich

**Affiliations:** a Department of Radiation Oncology and Radiation Therapy, University Medical Center of the Johannes Gutenberg University Mainz, Mainz, Germany; b Institute of Medical Biostatistics, Epidemiology and Informatics (IMBEI), University Medical Center of the Johannes Gutenberg University Mainz, Mainz, Germany; c Research Center for Immunotherapy (FZI), University Medical Center of the Johannes Gutenberg University Mainz, Mainz, Germany

**Keywords:** Head and neck cancer, Radiotherapy, Delayed elective nodal irradiation, Peripheral blood lymphocytes, DNA damage, Lymphopenia, Transcriptomics

## Abstract

**Background:**

Elective nodal irradiation (ENI) for locally advanced head-and-neck squamous cell carcinoma (LA-HNSCC) contributes to hematotoxicity and may impair radiotherapy-immunotherapy synergy. We investigated whether delayed ENI, using an upfront boost to gross disease and involved nodes followed by standard ENI, reduces radiation-induced lymphocyte DNA damage and lymphopenia (RIL) while modulating peripheral immune signaling compared with standard ENI.

**Materials and Methods:**

Twenty-eight LA-HNSCC patients assigned for adjuvant (*n* = 13) or definitive (*n* = 15) (chemo)radiotherapy were randomized to standard or delayed ENI (upfront boost: adjuvant: 2 × 2 Gy, *n* = 6/15; definitive: 5 × 2 Gy, *n* = 7/13). Blood was collected pre-treatment, 15 min and 24 h after the first fraction, and (definitive only) before fraction six and at treatment end. Primary endpoints: DNA damage in lymphocytes via γH2AX and 53BP1 foci (*n* = 25, pre-therapy, post-first and pre-sixth fraction) and dicentric chromosomes (*n* = 9, pre-therapy and pre-sixth fraction). Secondary endpoints: exploratory leukocyte transcriptomics (*n* = 4; pre-therapy, pre-sixth fraction and end of therapy), lymphocyte counts, and RIL (*n* = 28; pre-therapy and weekly during therapy).

**Results:**

Delayed ENI reduced initial planning target volume and whole-body dose by 9.9- and 4.4-fold (both *p* < 0.001), resulting in 3–4-fold lower DNA damage marker frequencies (*p* < 0.001) and fewer radiation-damaged lymphocytes (~11% vs. ~23%, *p* < 0.001). Baseline lymphocyte counts were preserved during the first treatment week, delaying RIL onset, whereas standard ENI induced an immediate decline in lymphocyte counts and RIL. Week 1 lymphocyte counts inversely correlated with planning target volume, whole-body dose, and DNA damage (all *p* < 0.01). Exploratory transcriptomic profiling revealed enhanced interferon and metabolic signaling with delayed ENI, whereas standard ENI preferentially activated inflammatory and damage-associated pathways.

**Conclusion:**

These findings suggest that delaying elective nodal irradiation may help preserve circulating immune cells during radiotherapy and provide a mechanistic rationale for future prospective studies evaluating whether this strategy can enhance radiotherapy–immunotherapy synergy in LA-HNSCC.

## Highlights

• First study of hematological effects of delayed elective nodal irradiation in HNSCC.
• Nodal sparing cut lymphocyte DNA damage and delayed radiation-induced lymphopenia.
• Transcriptomic data suggested antitumor immune signaling, without direct evidence.
• Delayed ENI mitigated hematotoxicity, warranting evaluation alongside immunotherapy.

## 1 Introduction

Besides technical innovations, the standard of care for locally advanced head and neck squamous cell carcinoma (LA-HNSCC) has remained unchanged for over two decades: resectable tumours are commonly managed by radical resection with neck dissection followed by adjuvant chemoradiotherapy (CRT), while unresectable disease receives definitive CRT, occasionally preceded by induction chemotherapy (CT) [1]. The addition of immune checkpoint inhibitors (ICIs) to curative-intent CRT has produced disappointing results in large phase III trials, including KEYNOTE-412 and JAVELIN-100 [2], [3], [4], [5], [6], [7]. Only recently, the KEYNOTE-689 trial established the first new standard of care in over 20 years: neoadjuvant pembrolizumab before surgery, followed by adjuvant CRT with continued ICI [8]. Also, the NIVOPOST-OP trial demonstrated improved disease-free survival with the addition of adjuvant nivolumab to CRT in high-risk resected LA-HNSCC [9].

RT can enhance the efficacy of ICIs by inducing immunogenic tumor cell death, thereby promoting local and distant antitumor immune responses [10]. However, RT inevitably damages the radiosensitive hematologic system and can abolish the effects of ICIs in combination [11]. Particularly, elective nodal irradiation (ENI) for LA-HNSCC has a significant negative impact on RT-ICI combinations [12], [13], [14], [15], [16]. Tumor-draining lymph nodes are key components of the cancer-immunity cycle, where tumor antigens are cross-presented and tumor-specific T cells undergo maturation [17]. The upfront or complete exclusion of elective nodal (EN) volumes can preserve RT-ICI synergy; however, this conclusion is based almost exclusively on data from preclinical models [12], [13], [14], [15], [16].

Also, radiation-induced lymphopenia (RIL) is a common side effect of HNSCC RT, linked to poorer outcomes and may compromise RT-ICI synergy [18]. To date, the interplay between ENI and RIL remains largely unexplored [19]. Besides simulations based on RT parameters to estimate dose to blood [20], biodosimetric markers of radiation-induced DNA damage in peripheral blood lymphocytes (PBLs) are a valuable tool for directly assessing blood exposure during RT [21].

In this study, we examined how delayed ENI in definitive and adjuvant CRT for LA-HNSCC patients influences radiation damage, hematotoxicity, and systemic immunogenic signaling, using radiation biomarkers in PBLs, RIL, and transcriptomic signatures in peripheral blood. The present study was designed as a translational investigation of radiobiological and immunogenic endpoints; it was not designed or powered to assess oncological efficacy or responsiveness, which therefore remain outside the scope of this manuscript and remain to be evaluated in future prospective trials.

## 2 Patients and methods

### 2.1 Study design and endpoints

This prospective, block-randomized controlled trial enrolled 28 patients with LA-HNSCC assigned to standard ENI or delayed ENI (upfront boost: adjuvant: 2 × 2 Gy; definitive: 5 × 2 Gy), followed by standard ENI during platinum-based CRT. The primary endpoint was the yield of radiation-induced DNA damage as γH2AX and 53BP1 foci (double-strand break surrogate markers) and dicentric chromosomes (DIC) in PBLs. Secondary endpoints included absolute and relative lymphocyte counts (ALCs and RLCs, respectively), incidence and grade of RIL, dosimetric parameters (planning target volume [PTV]; whole-body dose), and their correlations with biological markers, as well as exploratory longitudinal whole-blood transcriptomic profiling to assess immune pathway modulation.

### 2.2 Patients and therapy

We included patients with pathologically proven laryngeal, hypopharyngeal, p16-negative oropharyngeal, or oral cavity carcinoma. Patients who were either unsuitable for surgical resection or declined surgery were considered for definitive treatment. Following surgery, patients with either insufficient resection margins or involved lymph nodes with extracapsular extension (ECE) were included for adjuvant therapy. Treatment was determined by a multidisciplinary tumor board, and patients were enrolled after providing informed consent for RT and study participation. RT planning and treatment were performed in accordance with local standards. For definitive therapy, a 5 + 5 approach was used, while EN volumes were defined according to international standards [22]. In the adjuvant setting, volume and dose prescription also followed international standards [23]. Boost volumes were defined based on pathological risk factors and could include lymph node levels with ECE, areas with close surgical margins, or both. In the definitive cohort, cisplatin was administered weekly at 40 mg/m^2^ body surface area. In the adjuvant setting, cisplatin was administered at 20 mg/m^2^ body surface area on five days in week one and week five of RT. An overview of the different treatment regimens and dose distributions for standard ENI versus delayed ENI is shown in Fig. 1. Both standard and time-adjusted equivalent dose in 2 Gy fractions (EQD2) values [24] for different treatment schedules are provided in Supplementary Table 1. Ethical approval was obtained from the Ethics Committee of the Rhineland-Palatinate Chamber of Physicians (No. 2024–17,401 and 2024–17,191), and the study was registered in the German Clinical Trials Register (ID: DRKS00034748 and DRKS00034747). All donors provided informed consent, and the research was conducted in accordance with the Declaration of Helsinki. RT was performed by 6 MeV volumetric modulated arc therapy with a Clinac DHX, Unique™^,^ or Truebeam® (Varian Medical Systems, Palo Alto, CA, US) at the Department of Radiation Oncology and Radiation Therapy at the University Medical Center Mainz, Germany. Treatment planning was conducted with the treatment planning system “Eclipse” (Varian Medical Systems, Palo Alto, CA, USA). Each patient's whole-body dose (WBD), as the average dose per voxel over the whole-body volume, was calculated as described previously [21].

**Fig. 1:**
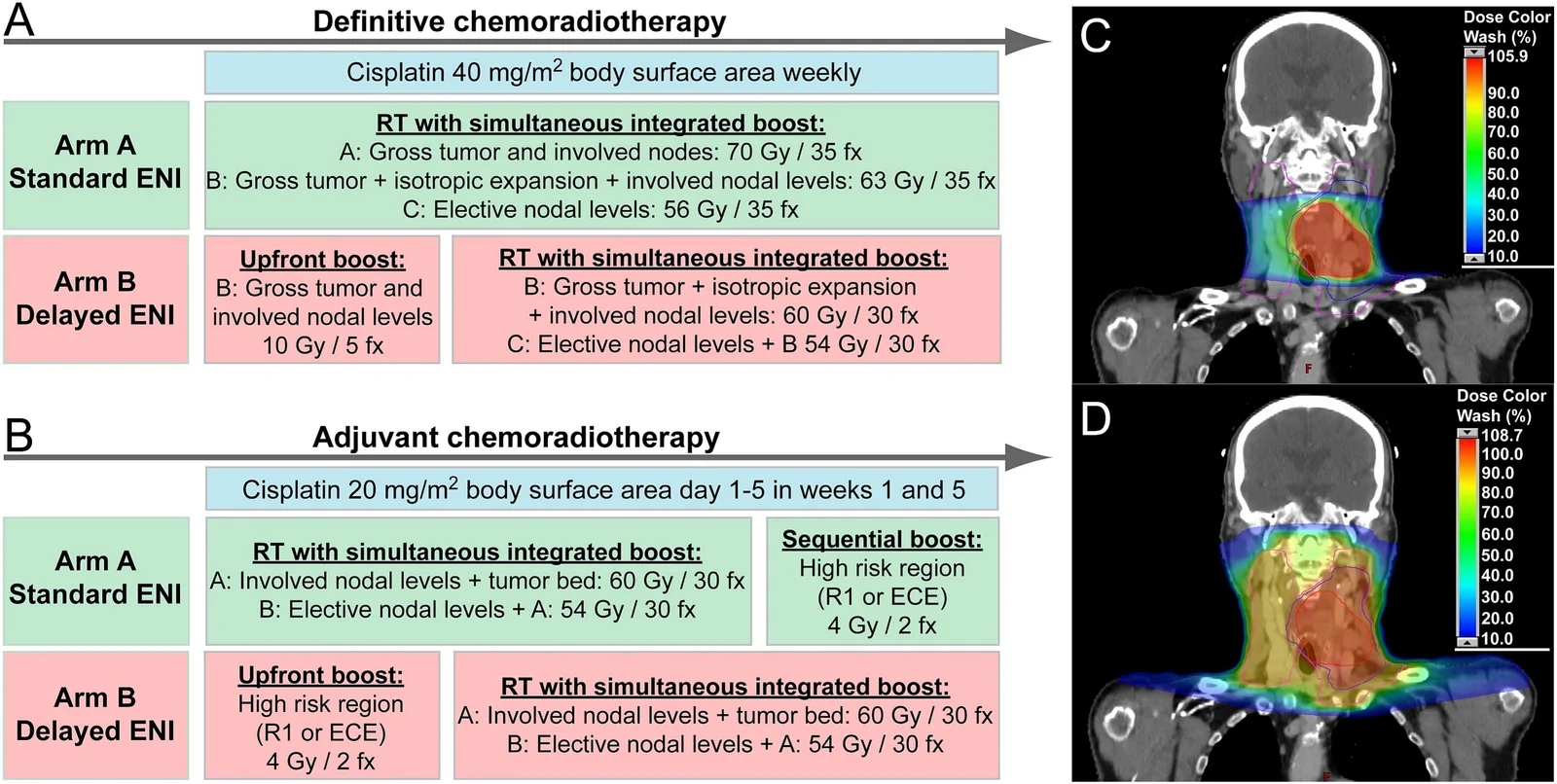
Treatment regimens for (A) definitive chemoradiotherapy (CRT) and (B) adjuvant CRT. At an alpha/beta of 10, doses were considered nearly isoeffective for definitive CRT patients using the equivalent dose in 2 Gy fractions (EQD2). Treatment planning images in dose color wash show the same patient with a hypopharyngeal tumor in the delayed elective nodal irradiation (ENI) arm: (C) Delayed ENI, and (D) after switching to standard ENI after 5 fractions. Planning target volumes are delineated as magenta: elective nodal levels; blue: high-risk nodal levels + primary tumor; red: macroscopic tumor. R1: positive surgical margins; ECE: extracapsular extension. (For interpretation of the references to color in this figure legend, the reader is referred to the web version of this article.)

### 2.3 Blood sampling and ex vivo irradiation

Blood was collected immediately before (pre-CRT), 15 min and 24 h after the first fraction of RT. In the definitive cohort, another sample was taken before the sixth fraction, and for transcriptomic analyses at the end of treatment (end-CRT). Blood collection and analysis procedures are summarized in Supplementary Fig. 1. A fraction of pre-CRT blood samples was irradiated ex vivo with 0.5 Gy as previously described [20] and analyzed for γH2AX and 53BP1 foci at 0.5 h post-irradiation.

### 2.4 γH2AX and 53BP1 focus quantification

The γH2AX and 53BP1 focus assay in PBLs has been performed as described previously [21], [25]. In unirradiated pre-CRT samples and all post-CRT samples, at least 500 cells were evaluated. 0.5 h after ex vivo irradiation of pre-CRT samples with 0.5 Gy, at least 50 cells were analyzed. Radiation-induced foci (RIF) were quantified by subtracting foci in unirradiated pre-CRT samples from total post-irradiation values. Representative microscopic images of γH2 AX and 53BP1 foci in PBLs are shown in Supplementary Fig. 2 A.

### 2.5 Dicentric assay.

The DIC assay was performed on blood samples from a subset of 9 patients with definitive therapy obtained pre-CRT and before the sixth fraction of RT, as described previously [21]. On average, 405 ± 138 (range: 203–631) metaphases were analyzed per sample. Basal DIC yields were subtracted from yields after RT.

### 2.6 Lymphocyte counts and lymphopenia.

ALCs were collected pre-CRT and weekly during CRT. The National Cancer Institute's Common Terminology Criteria for Adverse Events version 5.0 (CTCAEv5.0) was used to classify lymphopenia as grade 0/normal: >1000/mm^3^, grade 1: 800–999/mm^3^, grade 2: 500–799/mm^3^, grade 3: 200–499/mm^3^, and grade 4: <200/mm^3^ [26]. RLCs were calculated relative to the pre-CRT baseline.

### 2.7 Blood transcriptomics.

#### 2.7.1 Samples and RNA sequencing

Total RNA from leukocytes was collected from two patients each with standard ENI or delayed ENI in the definitive cohort, pre-CRT, pre-sixth fraction, and at end-CRT, as described previously [27]. One pre-CRT sample was unavailable for one patient with delayed ENI. Bulk RNA library preparation and transcriptome sequencing were conducted by Novogene (Novogene GmbH, Munich, Germany) according to the company's protocols, and quality control was performed as reported previously [28].

#### 2.7.2 Differential gene expression and gene set enrichment analysis

Differential expression analysis was performed using the limma-voom framework on TMM-normalized counts after low-count filtering, retaining 19,129 genes [29]. Genes on the X and Y chromosomes were removed before analysis to mitigate confounding by donor sex, which was collinear with treatment arm assignment in this cohort; pseudoautosomal region genes were retained given their autosomal expression behaviour. To compare longitudinal gene activity changes during treatment between the two arms, we used a statistical difference-in-differences (DiD) approach. For the delayed versus standard ENI arm, we measured how gene activity changed from before to after treatment, and then compared these two changes against each other: Delayed ENI Δ (post-treatment − pre-treatment) − standard ENI Δ (post-treatment − pre-treatment). Since each treatment arm was compared to its own pre-treatment baseline, this approach focused specifically on treatment-related effects rather than on pre-existing differences between groups. Moderated t-statistics were computed via empirical Bayes shrinkage. Gene set enrichment analysis (GSEA) on the moderated t-statistic ranking was used as the primary analytical output. GSEA was performed against the MSigDB Hallmark and Reactome collection. False discovery rate (FDR) < 0.05 was applied as the pathway-level significance threshold. Positive normalized enrichment scores (NES) in DiD contrasts indicate greater enrichment in the delayed ENI arm.

### 2.8 Sample size calculation, foci and DIC data and statistical analyses

Sample size estimation for primary DNA-damage (γH2AX foci) endpoints was based on our previously published biodosimetric data [21] with G*Power [30] using a two-sided unpaired *t*-test (α = 0.05, power = 80%) for mean γH2AX foci 15 min after the first RT fraction in PBLs of prostate cancer patients without (*n* = 25; 0.36 ± 0.18 foci/PBL) or with pelvic nodal irradiation (*n* = 6; 1.65 ± 0.33 foci/PBL). Based on an effect size of Cohen's d = 4.9, the required sample size was 1 and 5 patients per group (actual power = 90%), respectively. Prostate cancer patients showed a mean 5.7-fold difference in PTV between RT with (1431 ± 262 cm^3^) versus without (252 ± 101 cm^3^) pelvic nodal irradiation. Preliminary treatment-planning analyses in three HNSCC patients indicated an even higher mean 7.5-fold difference in PTV for the delayed ENI concept (140 ± 43 cm^3^) versus standard ENI (1055 ± 253 cm^3^), anticipating a larger effect size in the HNSCC setting. Sample sizes were conservatively increased to >5 patients per arm per cohort for this study. Accordingly, the study is powered for the primary DNA-damage endpoints only. Lymphocyte counts, RIL incidence and grade, transcriptomic profiles, and any oncologic endpoints reported in this manuscript are exploratory and hypothesis-generating and were not powered for confirmatory conclusions.

Summarized average yields and data are provided as the mean and standard deviation (SD) unless stated otherwise. Data handling, plotting, and statistics were done using SigmaPlot (version 14, Systat Software, USA) and QtiPlot (version 5.12.8, IONDEV SRL, ROU). The relationship between two variables was analyzed using the Pearson test. For comparison of the means of two or more groups, the Student's *t*-test or the one-way analysis of variance (ANOVA) was used, respectively. The chi-square test was used to evaluate associations between categorical variables. All levels of significance were set to α = 0.05. Follow-up was calculated using the reverse Kaplan–Meier method, starting from study inclusion.

Given the randomized design, formal baseline comparisons between treatment arms were not powered for confirmatory inference owing to the small sample size (*n* = 28). Nevertheless, baseline characteristics were compared between standard and delayed ENI using Wilcoxon rank sum test or Fisher's exact test for categorical variables. These comparisons are reported in Table 1 as descriptive measures of balance rather than as confirmatory tests. Multivariable adjustment was not pursued for the primary or secondary endpoints, as the number of covariates relative to the sample size precludes stable estimation.

**Table 1:** Patient demographics.

Characteristic	Adjuvant, *N* = 13			Definitive CRT, *N* = 15		
	Delayed ENI N = 7^1^	Standard ENI N = 6^1^	p-value^2^	Delayed ENI N = 6^1^	Standard ENI N = 9^1^	p-value^2^
Age at RT			0.5			0.8
Mean (SD)	64 (10)	60 (7)		64 (13)	61 (13)	
Median [Q1, Q3]	63 [59, 73]	62 [52, 64]		63 [58, 71]	62 [51, 70]	
Sex			>0.9			0.2
Female	2 (29%)	1 (17%)		3 (50%)	1 (11%)	
Male	5 (71%)	5 (83%)		3 (50%)	8 (89%)	
Karnofsky Performance Score before RT			0.6			>0.9
KPS < 70	1 (14%)	2 (33%)		0 (0%)	1 (11%)	
KPS 70+	6 (86%)	4 (67%)		6 (100%)	8 (89%)	
Age-adjusted Charlson Comorbidity Index			0.8			0.9
Mean (SD)	2.14 (1.21)	3.17 (2.40)		2.50 (2.07)	2.78 (2.44)	
Median [Q1, Q3]	2.00 [1.00, 3.00]	3.00 [1.00, 5.00]		2.50 [1.00, 3.00]	3.00 [1.00, 4.00]	
Location of primary tumor			0.5			0.6
Hypopharynx	0 (0%)	0 (0%)		1 (17%)	1 (11%)	
Larynx	1 (14%)	2 (33%)		1 (17%)	1 (11%)	
Nasal cavity	0 (0%)	0 (0%)		1 (17%)	0 (0%)	
Oral Cavity	2 (29%)	0 (0%)		1 (17%)	0 (0%)	
Oropharynx	0 (0%)	2 (33%)		2 (33%)	6 (67%)	
Salivary gland	1 (14%)	0 (0%)		0 (0%)	0 (0%)	
Tongue	3 (43%)	2 (33%)		0 (0%)	1 (11%)	
T-Stage (AJCC 8th edition)			0.6			0.5
2	1 (14%)	2 (33%)		2 (33%)	1 (11%)	
3	4 (57%)	4 (67%)		2 (33%)	6 (67%)	
4	2 (29%)	0 (0%)		2 (33%)	2 (22%)	
N-Stage (AJCC 8th edition)			>0.9			0.6
0	3 (43%)	2 (33%)		2 (33%)	2 (22%)	
1	0 (0%)	0 (0%)		2 (33%)	1 (11%)	
2	2 (29%)	1 (17%)		2 (33%)	5 (56%)	
3	2 (29%)	3 (50%)		0 (0%)	1 (11%)	
Extracapsular extension			>0.9			>0.9
ECE	3 (43%)	3 (50%)		–	1 (11%)	
No ECE	1 (14%)	1 (17%)		–	–	
No neck dissection	–	–		6 (100%)	8 (89%)	
No pathologically involved nodes	3 (43%)	2 (33%)		–	–	
Surgical margins			>0.9			>0.9
Insufficient Margins or R1	5 (71%)	4 (67%)		–	1 (11%)	
No tumor resection	–	–		6 (100%)	8 (89%)	
Sufficient margins	2 (29%)	2 (33%)		–	–	
Smoking Status			0.4			>0.9
Former Smoker	1 (14%)	3 (50%)		2 (33%)	3 (33%)	
Never-Smoker	2 (29%)	0 (0%)		1 (17%)	1 (11%)	
Smoker	4 (57%)	3 (50%)		3 (50%)	5 (56%)	

1 n (%); ^2^Wilcoxon rank sum test, Fisher's exact test; RT, radiotherapy; SD, standard deviation; AJCC, American Joint Committee on Cancer; ECE, extracapsular extension; R1, microscopic residual tumor.

## 3 Results

### 3.1 Patient and treatment characteristics.

In total, 28 patients designated to either adjuvant or definitive treatment were included between April 2024 and December 2025 and were block randomized into two groups of delayed or standard ENI. 15 patients were treated with definitive CRT (standard ENI: *n* = 9, delayed ENI: *n* = 6), and 13 patients were treated with adjuvant RT or CRT (standard ENI: *n* = 6, delayed ENI: *n* = 7). RT was discontinued prematurely in one case due to patient preference; all other patients received full-dose RT. Concurrent CRT was administered in 22 patients, with 6 of 13 patients in the adjuvant arm receiving RT only (standard ENI: *n* = 3, delayed ENI: *n* = 3). One patient in the definitive arm received weekly docetaxel due to an advanced age of over 80 [31]. All other patients received cisplatin, and 13 patients received a cumulative cisplatin dose of at least 200 mg/m^2^ body surface area. Median follow-up was 10.97 months (95% confidence interval (CI): 8.42–17 months). 12-month overall and progression-free survival were 96.0% (95% CI: 88.6–100%) and 80.7% (95% CI: 64.9–100%), respectively. Patient characteristics are summarized in Table 1. This study was not statistically powered to detect differences in toxicity between treatment arms. Accordingly, given the limited sample size, no clinically relevant differences in the incidence, severity, or pattern of acute or late toxicities were observed between the standard and delayed ENI groups, either in the overall cohort or within the adjuvant and definitive treatment subgroups. A complete event-level listing, stratified by treatment arm and grade, is provided in Supplementary Table 2.

### 3.2 DNA damage in peripheral blood lymphocytes.

Details on baseline DNA damage and ex vivo radiosensitivity are provided in Supplementary Fig. 2B**.** Fifteen minutes after the first RT fraction, RIF levels in PBLs correlated strongly with PTV size and WBD (Fig. 2A). Individual and averaged data for DNA damage markers and RT parameters are provided in Supplementary Table 3 and Supplementary Fig. 3. The EN-sparing boost used for delayed ENI reduced the initial PTV and WBD by 9.9- and 4.4-fold, respectively (both *p* < 0.001), resulting in an approximately fourfold lower mean RIF frequency per PBL than standard ENI (*p* < 0.001). After 24 h, overall RIF frequencies declined because of DNA repair and lymphocyte recirculation; however, residual RIF frequencies remained moderately correlated with PTV and WBD and were approximately threefold lower in the delayed ENI group than in the standard ENI group (Fig. 2B; *p* < 0.01). A pre-specified subgroup analysis stratifying patients by definitive versus adjuvant CRT is provided in Supplementary Fig. 4 and showed similar results across both subgroups.

**Fig. 2:**
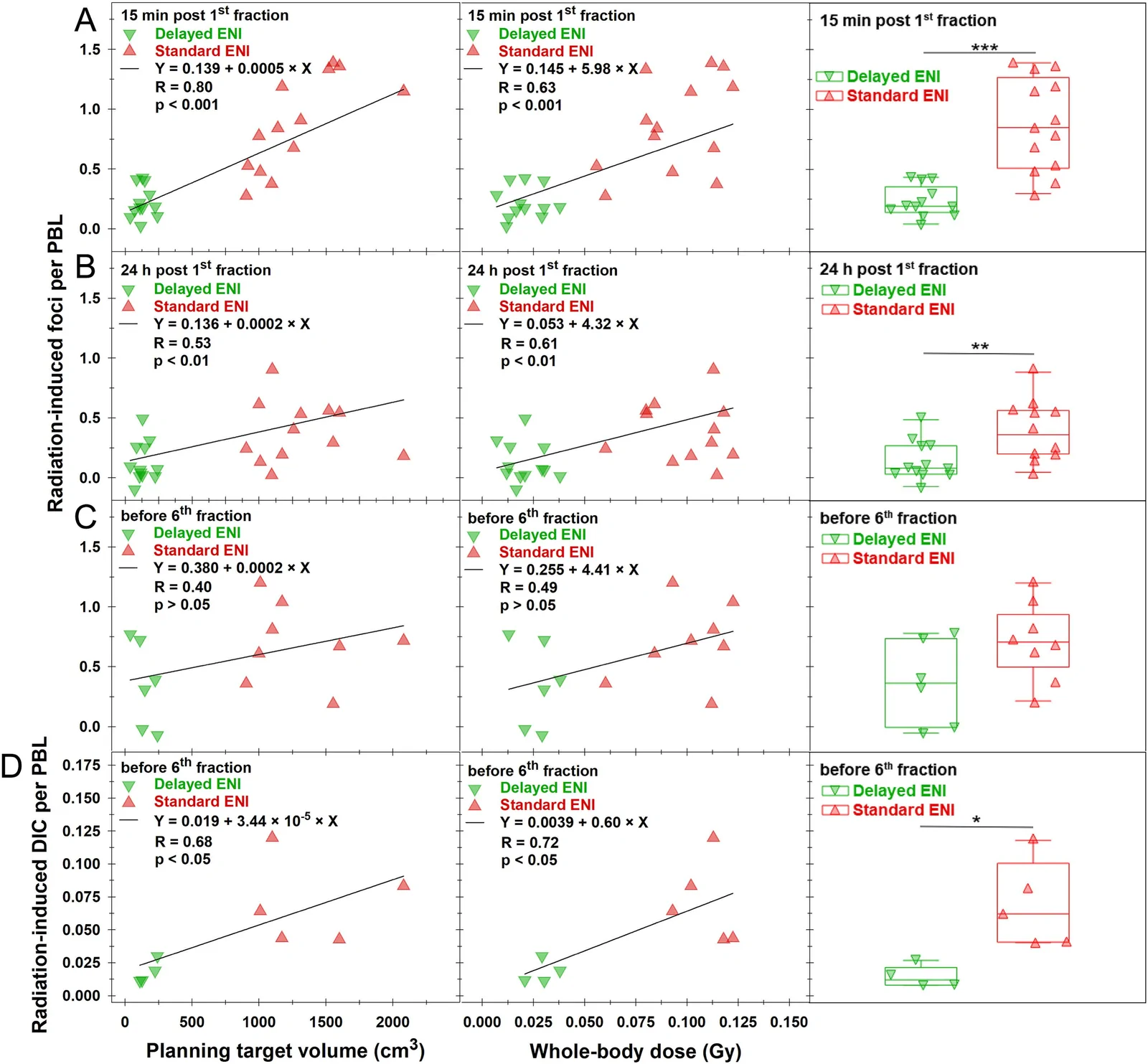
Radiation-induced (A-C) γH2AX/53BP1 foci and (D) dicentric chromosomes (DIC) in patients' peripheral blood lymphocytes (PBLs) after radiotherapy. Data are shown relative to the planning target volume (left panel), whole-body dose (middle panel), and stratified by standard elective nodal irradiation (ENI) and delayed ENI (right panel): (A) 15 min after the first fraction, (B) 24 h after the first fraction, and (C, D) before the sixth fraction (definitive only). The lines in the left and middle panels represent linear fits to the data, along with the corresponding function, Pearson correlation coefficient (R), and the *p*-value of the fit. Statistical comparison between delayed and standard ENI (right panel) was conducted using Student's *t*-test (* *p* < 0.05; ** *p* < 0.01; *** *p* < 0.001).

In 14 patients receiving definitive CRT, RIF levels were measured before the sixth fraction to assess cumulative DNA damage before the delayed ENI arm transitioned to standard ENI (Fig. 2C). RIF per PBL showed non-significant, moderate correlations with PTV and WBD, with about twofold higher residual RIF after standard ENI than delayed ENI (*p* > 0.05). RIF accumulation in PBLs during RT is influenced by DNA repair, fractionation intervals, and treatment interruptions. Therefore, the DIC assay was used as a more reliable measure of cumulative RT-induced DNA damage [21]. DIC frequencies before the sixth fraction correlated strongly with PTV and WBD and were significantly about fourfold lower after delayed versus standard ENI (Fig. 2D; *p* < 0.05).

The proportion of radiation-damaged PBLs (≥1 RIF per PBL) 15 min after the first fraction also correlated strongly with PTV size and WBD (Fig. 3), and delayed ENI reduced the proportion of radiation-damaged PBLs about twofold compared with standard ENI (10.9 ± 6.5% versus 23.3 ± 9.8%; *p* < 0.001). Standard ENI also generated significantly more PBLs harboring multiple RIF (2–15 per cell; Supplementary Fig. 5). At 24 h after the first fraction, and before the sixth fraction, correlations with PTV and WBD were weak, and differences between treatment groups were no longer significant (Supplementary Fig. 5; *p* > 0.05).

**Fig. 3:**
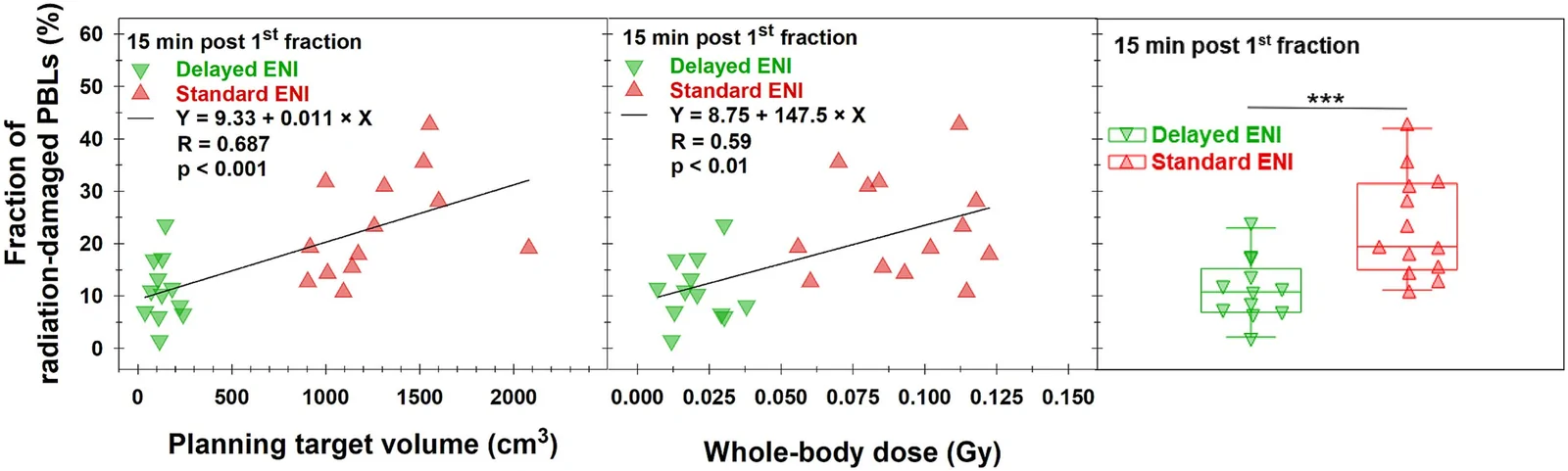
The fraction of radiation-damaged peripheral blood lymphocytes (PBLs) after radiotherapy. The fraction of PBLs with ≥1 radiation-induced γH2AX/53BP1 focus relative to the planning target volume (left panel), whole-body dose (middle panel), and stratified by standard elective nodal irradiation (ENI) and delayed ENI (right panel) 15 min after the first fraction. The lines in the left and middle panels represent linear fits to the data, along with the corresponding function, Pearson. Correlation coefficient (R), and the *p*-value of the fit. Statistical comparison between delayed ENI and standard ENI was conducted using Student's *t*-test (* *p* < 0.05; ** *p* < 0.01; *** *p* < 0.001).

### 3.3 Lymphocyte counts and lymphopenia.

After the first week of therapy, delayed ENI preserved ALCs at baseline in both adjuvant and definitive cohorts, while standard ENI caused an immediate decline (Fig. 4A). After adaptation to standard ENI, the delayed ENI arm also showed a marked ALC decrease in week 2. All patients developed RIL, but onset differed significantly between treatment arms (χ^2^ = 9.78, df = 4, *p* = 0.04; Supplementary Table 4): RIL first occurred after week one with standard ENI and after week two with delayed ENI. By week two, RIL incidence (58% versus 61%, 7/12 versus 7/13) and subsequent progression of RIL were comparable between treatment arms. Nadir RIL severity also did not differ significantly (χ^2^ = 2.70, df = 3, *p* = 0.44), although grade IV RIL was more frequently observed with standard ENI (21% versus 8%, 3/14 versus 1/13, *p* > 0.05).

**Fig. 4:**
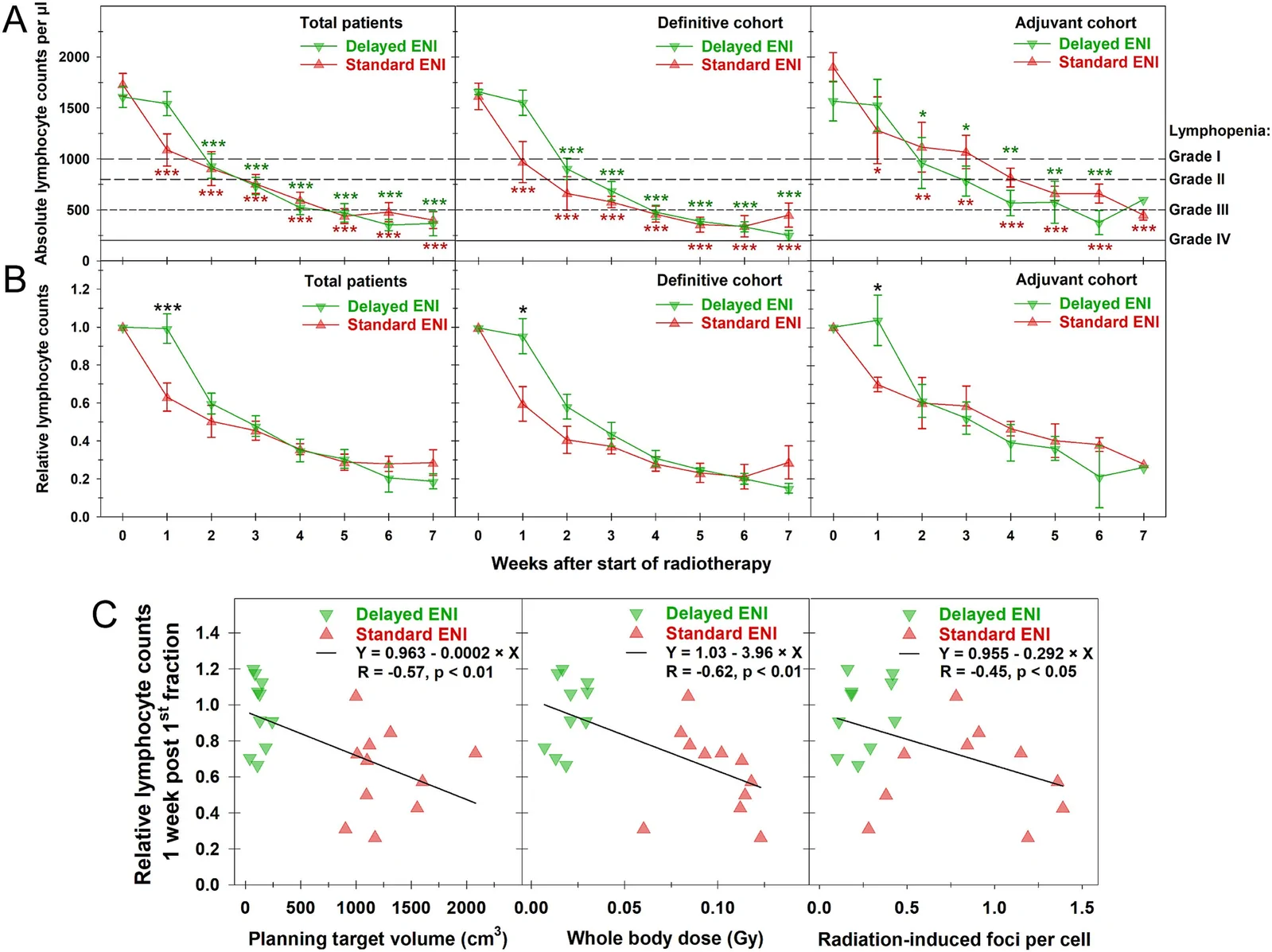
Lymphocyte counts during therapy. (A) Absolute or (B) relative lymphocyte counts (RLCs) for all patients (left panel) and for patients receiving definitive (middle panel) or adjuvant (right panel) therapy stratified by standard elective nodal irradiation (ENI) and delayed ENI. Grades of lymphopenia per CTCAE v5 are indicated. Data are presented as the mean and the standard error of the mean. The patient numbers for each data point are in Supplementary Table 4. Statistical comparison between (A) before and during therapy within each treatment group or (B) between delayed and standard ENI was conducted by One-Way ANOVA or Student's *t*-test, respectively (* *p* < 0.05; ** *p* < 0.01; *** *p* < 0.001). (C) Correlations of relative lymphocyte counts per patient after one week of chemoradiotherapy and the planning target volume, whole-body dose, and radiation-induced foci per cell 15 min after the first fraction stratified by elective nodal irradiation status. The lines represent linear fits to the data, along with the corresponding function, Pearson correlation coefficient (R), and the *p*-value of the fit.

Due to inter-patient variability in ALCs, RLCs were used for comparisons between delayed and standard ENI (Fig. 4B). After week one of treatment, RLCs remained stable and were significantly higher with delayed ENI than with standard ENI, overall and after stratification by definitive or adjuvant treatment. Individual ALC and RLC data are provided in Supplementary Table 5. Week one RLCs were inversely correlated with PTV, WBD, and RIF per PBL 15 min after the first fraction (Fig. 4C).

### 3.4 Leukocyte transcriptome profiling.

Leukocyte bulk RNA-seq was conducted to characterize systemic molecular responses as part of an exploratory, hypothesis-generating analysis in a preliminary, limited sample size per treatment arm (*n* = 2). Before therapy, transcriptomic differences between treatment arms were minimal, confirming baseline comparability. Pre-sixth fraction, DiD analysis showed greater enrichment of lymphocyte metabolic and proliferative programs with delayed ENI, including oxidative phosphorylation, E2F and MYC targets, DNA repair, and glycolysis (all FDR < 0.001; Fig. 5A). In contrast, standard ENI preferentially enriched TNF-α/NF-κB signaling (NES = −1.52, FDR = 0.012), consistent with acute inflammatory responses. At treatment end, the delayed ENI group remained enriched for IFN-α/IFN-γ signaling, oxidative phosphorylation, and MYC targets (all FDR ≤ 0.001); Standard ENI promoted enrichment of mitotic spindle, TGF-β, and IL6/JAK/STAT3 pathways.

**Fig. 5:**
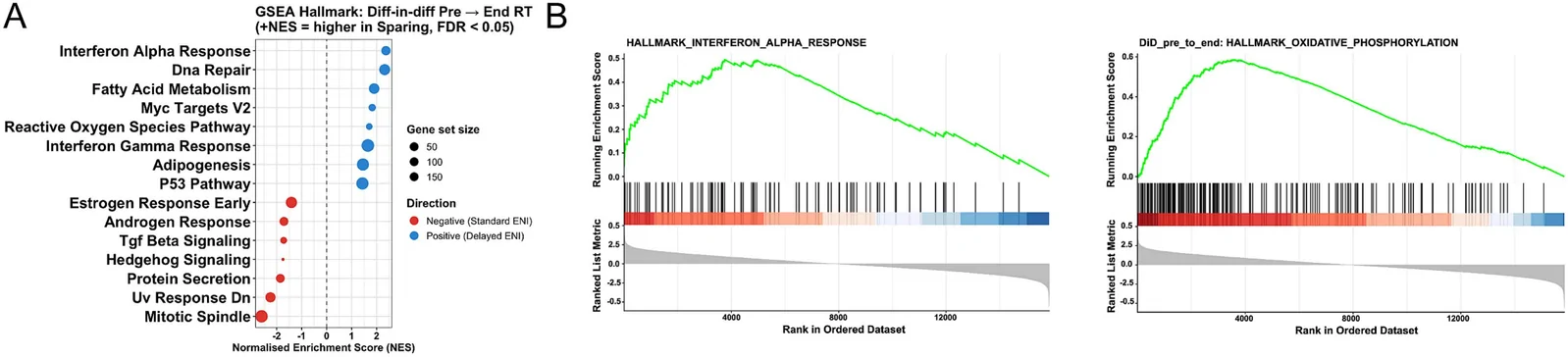
The transcriptomic response in whole blood during therapy. (A) Gene set enrichment analysis (GSEA) comparing the pre-therapy to end-therapy trajectory in delayed elective nodal irradiation (ENI) versus standard ENI. Only pathways reaching FDR < 0.05 are shown. Positive normalized enrichment scores (NES) indicate greater enrichment in the delayed ENI arm; negative NES indicate greater enrichment in the standard ENI arm. (B) GSEA barcode plots for Interferon Alpha Response (NES = +2.39, FDR < 0.001), enriched in the delayed ENI arm, and Mitotic Spindle (NES = −2.61, FDR < 0.001), enriched in the standard ENI arm. The ranked list metric (bottom panel) reflects the moderated t-statistic from the difference-in-differences contrast.

Within-arm analysis confirmed divergent trajectories: delayed ENI induced robust IFN-α/IFN-γ signaling (both FDR < 0.001), whereas standard ENI was dominated by inflammatory and TNF-α signaling without significant IFN-α induction (Fig. 5B). At treatment end, cross-sectional comparison confirmed that the delayed ENI arm remained enriched for IFN-α response and oxidative phosphorylation. The standard ENI arm showed greater inflammatory, TNF-α, and mitotic spindle enrichment (Supplementary Table 6). At the gene level, delayed ENI specifically upregulated ISG15, supporting IFN-α pathway activation, and PARP10, consistent with enhanced MYC, E2F, and DNA repair programs in proliferating lymphocytes (Supplementary Fig. 6).

## 4 Discussion

This study examined how delayed ENI in definitive and adjuvant CRT for LA-HNSCC patients influences hematotoxicity and systemic immunogenic signaling, using radiation biomarkers and preliminary transcriptomic analysis in peripheral blood and RIL. To our knowledge, this is the first study to explore delayed ENI strategies in LA-HNSCC across both treatment settings with these methods.

For DNA damage markers in PBLs, we found good to very good correlations with PTV and the WBD for RIF up to 24 h after the first fraction and for DIC before the sixth fraction, consistent with our prior findings in other tumor types [21], [32]. This led to a significant reduction in the mean DNA damage frequencies per PBL due to delayed ENI with smaller PTVs and lower WBDs during the early boost phase. Also, the fraction of radiation-damaged PBLs with RIF immediately after the first fraction correlated strongly with both PTV and WBD. With delayed ENI, we observed a significantly lower average proportion of 13% radiation-damaged PBLs per RT fraction, with significantly lower counts of RIF per PBL compared to standard ENI. Overall, given the high radiosensitivity of PBLs [25], [33], a higher rate of apoptosis can be expected with standard ENI, in line with the immediate drop in ALCs after the start of treatment. A significant negative correlation between RLCs and the rates of RIFs in PBLs also confirmed this, indicating that RIF in PBLs after RT could serve as a rapid, predictive biomarker for RIL severity. Previously, only Lee et al. [34] showed a moderate correlation between the fraction of PBLs with chromosomal aberrations and the nadir of normalized white blood cell and lymphocyte counts in 22 lung cancer patients at the end of carbon-ion RT. However, RIL was generally mild, likely due to normal tissue sparing with particle RT. Due to the small patient sample, we found no correlation between DIC yields in PBLs or the fraction of aberrant PBLs and RLCs after the first week of CRT.

In the present study, all patients developed RIL during CRT. While about 40% of standard ENI patients showed RIL after week one of CRT, it did not appear in the delayed ENI group until week two after the adjustment to standard ENI. Surprisingly, even in the adjuvant cohort, where delayed ENI was limited to a two-fraction upfront boost, lymphocyte counts remained stable, suggesting a sustained immune-spring effect initiated very early during RT. However, after the introduction of ENI in the delayed ENI arm, ALCs declined rapidly, indicating increased radiosensitivity following acute sublethal damage during the initial phase of therapy. A longer delayed ENI interval, as used in the definitive setting (five daily fractions plus two weekend days), may be immunologically preferable, as effective T-cell priming, clonal expansion, and tumor infiltration require approximately 3–7 days [35]. By week three, nearly all patients in both arms had developed RIL. Although grade 4 RIL was more frequent with standard ENI, the small sample size limited statistical power. However, these findings suggest that delayed ENI may attenuate severe immunotoxicity after RIL onset, but the observations on RIL kinetics and nadir severity are hypothesis-generating. They do not constitute evidence of reduced clinical toxicity, and the trend toward more frequent grade 4 RIL with standard ENI requires confirmation in larger cohorts before any clinical interpretation can be drawn.

Only a few studies have evaluated the impact of ENI de-escalation, which has generally not been linked to an increased risk of nodal recurrences [36], on RIL in HNSCC, reporting less grade 4 RIL at nadir and up to 9 months after RT with reduced ENI [19]. However, direct comparison with our findings is limited by the fundamentally different nodal-sparing strategy using delayed ENI. RIL has been proposed as a key contributor to the limited efficacy of RT-ICI combinations in HNSCC, alongside ENI, the lack of predictive biomarkers, and RT-induced immunosuppression within the tumor microenvironment [37], [38], [39], [40], [41], [42]. Although baseline ALCs generally do not predict ICI response, persistent RIL is consistently associated with poor outcomes. Moreover, curative CRT disrupts lymphoid–myeloid crosstalk in HNSCC patients, driving sustained immune remodeling that may impair antitumor immunity and reduce ICI efficacy [43].

To identify such therapy-associated alterations in immune signaling pathways and potential biomarkers for therapeutic responses, we performed blood transcriptomic profiling. Given the small number of patients per treatment arm, these analyses were intended as exploratory and hypothesis-generating rather than confirmatory, and the findings reported here should be interpreted with caution. Despite being limited to only two patients per arm from the definitive cohort, DiD analysis revealed distinct immune responses within the first week of therapy and divergent longitudinal development between treatment arms. The acute metabolic activation observed with delayed ENI is consistent with preservation of ALCs. In contrast, standard ENI induced an acute damage-associated inflammatory program, likely driven by innate myeloid and stromal responses rather than adaptive immunity, in line with the early drop in ALCs [44].

By the end of treatment, the delayed ENI arm was characterized by transcriptomic signatures of type I/II IFN signaling, pathway-level patterns that are consistent with, but do not directly demonstrate, antigen-driven lymphocyte activation followed by effector T-cell expansion, biological processes commonly associated with antitumoral immune responses [45]. In contrast, the standard ENI arm showed increased inflammatory, TNF-α, mitotic spindle, and myeloid-associated signaling, pathway signatures that are compatible with, rather than direct evidence of, emergency myelopoiesis and chronic myeloid activation associated with tissue damage and loss of blood cells [46], [47]. Together, the key (preliminary) findings were a divergence between IFN-related pathway signatures in the delayed ENI arm, patterns commonly linked to antiviral and antitumor immunity, and damage-related pathway signatures in the standard ENI arm, patterns commonly linked to sterile inflammation. Notably, these divergent transcriptional programs emerged despite similar RIL trajectories following standard ENI adaptation, suggesting that the IFN-related pathway enrichment observed with delayed ENI may reflect, but does not prove, preservation of early antitumor immune activation beyond its effects on circulating lymphocyte counts. These exploratory observations provide a strong rationale for further investigating the effects of delayed ENI on immune cell subpopulations and immunogenic signaling to enhance RT–ICI synergy in LA-HNSCC in adequately powered prospective studies.

Our study has several limitations. First, the median follow-up to date was less than one year. Any claims regarding improved locoregional control, recurrence risk in initially unirradiated nodal stations, or enhanced ICI responsiveness with delayed ENI remain speculative and are not supported by the present data. In addition, adverse events were not systematically collected due to the study's radiobiological focus. Second, sex was not used as a stratification factor, resulting in an imbalance between study arms (delayed ENI: 71.4% of women [5/7] versus 38.1% of men [8/21]). Although this is unlikely to have affected results such as the induction of DNA damage foci [21], confounding effects cannot be excluded. Sex- and gender-related differences in innate and adaptive immune responses [48], in RT-related responses and toxicity [49], and in combination with ICIs [50] have been reported, and future studies should incorporate sex as a prespecified stratification factor. Of note, although the study used block randomization, formal baseline comparisons between treatment arms were performed descriptively rather than confirmatorily, as the sample size does not provide adequate power to detect modest baseline differences. No statistically significant baseline imbalances were observed between arms; however, this finding reflects limited statistical power rather than guaranteed balance, and residual confounding cannot be excluded.

Third, not all patients received concurrent cisplatin, with omissions occurring almost exclusively in the adjuvant cohort. However, an effect on the biological endpoints can be ruled out, as cisplatin was initiated only after the second RT fraction, and all patients in the definitive cohort subsequently received the same regimen. Although CT is often considered a major contributor to lymphopenia, our data suggest that RT is the predominant driver. Consistent with this, our previous ex vivo studies in isolated PBLs demonstrated only a modest additive effect of cisplatin on radiobiological endpoints compared with the substantially greater radiation-induced effects, such as apoptosis [25]. Likewise, within the adjuvant cohort, the course of RIL was similar regardless of concurrent CT, although subgroup sizes for comparisons were very small (Supplementary Fig. 7). Fourth, the transcriptomic analyses were exploratory in nature and based on a small sample size (*n* = 2 per arm from the definitive cohort). They were therefore not powered for definitive conclusions, and reported FDR values are descriptive rather than confirmatory. These data are intended to generate hypotheses regarding differential immune signaling between delayed and standard ENI, and validation in larger, independent cohorts with predefined immune endpoints will be required before any clinical or mechanistic conclusions can be drawn. Fifth, the inclusion of both adjuvant and definitive treatment cohorts represents a further limitation, as these settings differ in disease characteristics, target volumes, concomitant systemic treatment, and the extent and duration of delayed ENI. Given the small sample size, reliable assessment of treatment-by-cohort interactions was not feasible. Therefore, the findings should be considered hypothesis-generating, and future studies should evaluate these treatment settings separately.

## 5 Conclusion

Our study shows that delayed ENI in LA-HNSCC significantly reduces RT-induced acute DNA damage in PBLs and delays RIL, while preliminary transcriptomic profiles point toward a systemic immune signature suggestive of antitumor activity. The present data do not establish improved oncological outcomes, reduced clinical toxicity, or enhanced ICI efficacy with delayed ENI. Prospective validation in larger cohorts is highly warranted to confirm whether reduced nodal damage and transient peripheral lymphocyte preservation enhance T-cell priming, clonal expansion, antitumor memory, and ultimately, ICI efficacy and patient outcomes.

## Funding

This study was supported by the Bundesministerium für Forschung, Technologie und Raumfahrt (BMFTR, German Federal Ministry of Research, Technology and Space), Grant 02NUK084A, and by the 10.13039/501100001659Deutsche Forschungsgemeinschaft (DFG, German Research Foundation), Projektnummer 318,346,496-SFB1292/2TP19N.

## Declaration of competing interest

The authors declare the following financial interests/personal relationships which may be considered as potential competing interests: Sebastian Zahnreich reports financial support was provided by Federal Ministry of Research Technology and Space. Federico Marini reports financial support was provided by German Research Foundation. If there are other authors, they declare that they have no known competing financial interests or personal relationships that could have appeared to influence the work reported in this paper.

## Data Availability

Research data are stored in an institutional repository and will be shared on request to the corresponding author. Processed RNA-seq data are available at https://github.com/AhmedSAHassan/HNSCC_Transcriptomic_Dataset.
