# Supplementary material for: Delayed elective nodal irradiation in head-and-neck cancer radiotherapy reduces acute lymphocyte damage, lymphopenia, and modulates immune signatures
Source: Clin Transl Radiat Oncol. 2026 Oct 8;62:101315. doi: 10.1016/j.ctro.2026.101315 (PMC13675739; doi:10.1016/j.ctro.2026.101315)
Supplement: Supplementary file 1 [file mmc1.docx]

**Supplementary Material**

**Delayed Elective Nodal Irradiation in Head‑and‑Neck Cancer Radiotherapy Reduces Lymphocyte Damage, Lymphopenia, and Modulates Immune Signatures**

Justus Kaufmann^1^, Ahmed Salah^1,2^, Federico Marini^2,3^, Sophia Drabke^1^, Nina Gercek^1^, Stephanie Breinich^1^, Laura Oebel^1^, Heinz Schmidberger^1^, and Sebastian Zahnreich^1^

^1^Department of Radiation Oncology and Radiation Therapy, University Medical Center of the Johannes Gutenberg University Mainz, Mainz, Germany

^2^Institute of Medical Biostatistics, Epidemiology and Informatics (IMBEI), University Medical Center of the Johannes Gutenberg University Mainz, Mainz, Germany

^3^Research Center for Immunotherapy (FZI), University Medical Center of the Johannes Gutenberg University Mainz, Mainz, Germany

**Supplementary Table 1:** Time-dependent equivalent dose in 2 Gy fractions	2

**Supplementary Figure 1:** Study workflow and biospecimen collection	3

**Supplementary Figure 2:** Quantification of colocalizing γH2AX/53BP1 foci in peripheral blood lymphocytes (PBLs).	4

**Supplementary Table 2:** Treatment-related adverse events	5

**Supplementary Table 3:** Individual and summarized data on DNA damage markers	6

**Supplementary Figure 3:** Radiation-induced colocalizing γH2AX/53BP1 foci in peripheral blood lymphocytes post-CRT for all patients	7

**Supplementary Figure 4:** Radiation-induced γH2AX/53BP1 foci in peripheral blood 8 lymphocytes after definitive or adjuvant radiotherapy

**Supplementary Figure 5:** Analysis of the proportion of radiation-damaged peripheral blood lymphocytes	9

**Supplementary Table 4:** Timing and grade of radiation-induced lymphopenia	10

**Supplementary Table 5:** Individual and summarized data for absolute lymphocyte counts	11

**Supplementary Table 6:** Key pathway gene set enrichment analysis results across primary contrasts	12

**Supplementary Figure 6:** Normalised expression counts of ISG15 (left) and PARP10 (right) across treatment arms and timepoints.	13

**Supplementary Figure 7:** Relative lymphocyte counts one week post-1^st^ fraction in the adjuvant cohort stratified according to chemotherapy status.	14

# Supplementary Table 1

Time-dependent equivalent dose in 2 Gy fractions (EQD2) was calculated considering tumor repopulation starting with a delay of 28 days and 0.9 Gy for every day thereafter. EQD2 was calculated for both normal tissue (α/β= 3) and tumor (α/β = 10). For normal tissue, the dose could be considered almost identical, with less than 0.5 Gy difference in total dose. Time-dependent EQD2 suggests at least similar efficacy when considering tumor repopulation.

| Dose calculation | Standard ENI arm | Delayed ENI arm | Intermediate risk: Standard ENI arm | Intermediate risk: Delayed ENI arm |
| --- | --- | --- | --- | --- |
| EQD2 (α/β = 3) | 51.52 | 51.84 | 60.48 | 60 |
| EQD2 (α/β = 10) | 54.13 | 53.1 | 61.95 | 60 |
| Time dependent EQD2 (α/β = 10) | 39.88 | 45.47 | 53.05 | 60 |

ENI, elective nodal irradiation

# Supplementary Figure 1: Study workflow and biospecimen collection

*AI-generated, Gemini, Google,* [*https://gemini.google.com/*](https://gemini.google.com/)*, 07/24/2026.*

**Supplementary Information and Figure 2**

To assess basal DNA damage markers and radiation response in patient peripheral blood lymphocytes (PBLs), we measured basal γH2AX/53BP1 foci and dicentric chromosomes (DIC) in unirradiated pre-chemoradiotherapy (CRT) samples, and radiation-induced foci (RIF) 30 minutes after 0.5 Gy ex vivo irradiation. Individual levels of DNA damage in PBLs and raw data are provided in Supplementary Table 2. Basal γH2AX/53BP1 foci per PBL varied markedly among patients (0.37 ± 0.18, range: 0.04–0.88, CV = 48%), highlighting the need to account for variability after irradiation. After 0.5 Gy X-rays, mean RIF counts per PBL were similar after basal correction (4.40 ± 0.33, range: 3.67–4.93, CV = 7.4%), indicating comparable DSB induction across patients. Basal DIC were detected only in two of 9 analyzed patients scheduled for definitive CRT and were subtracted from post-CRT data.

**Supplementary Figure 2:** Quantification of colocalizing γH2AX/53BP1 foci in peripheral blood lymphocytes (PBLs). (A) Representative fluorescence microscopy image of basal (0 Gy) and ex vivo radiation-induced (0.5 Gy, 30 min) foci in PBLs as quantified (upper panel). Overview fluorescence image of foci in PBLs before and 15 minutes after the first radiotherapy fraction (lower panel). (B) Individual basal and ex vivo radiation-induced (0.5 Gy, 30 min) foci in PBLs collected before radiotherapy from each patient. Shown are total foci counts for 0 Gy and 0.5 Gy ex vivo and only radiation-induced foci (0.5 Gy ex vivo – 0 Gy).

**Supplementary Table 2**

Treatment-related adverse events (CTCAE v5.0) and weight loss, stratified by elective nodal irradiation strategy (delayed vs. standard) and treatment setting (definitive vs. adjuvant chemoradiotherapy).

|  | Delayed ENI, definitive CRT (n = 6) | Delayed ENI, adjuvant CRT (n = 7) | Standard ENI, definitive CRT (n = 9) | Standard ENI, adjuvant CRT (n = 6) |
| --- | --- | --- | --- | --- |
| **Weight loss** |  |  |  |  |
| Absolute weight loss, mean ± SD (kg) | 7.10 ± 2.25 | 4.97 ± 5.23 | 7.14 ± 5.73 | 8.38 ± 4.99 |
| Relative weight loss, mean ± SD (%) | 10.57 ± 2.55 | 6.85 ± 7.18 | 9.88 ± 7.73 | 10.57 ± 3.70 |
| **Mucositis** |  |  |  |  |
| Grade 0 | 0 | 0 | 1 | 0 |
| Grade 1 | 1 | 3 | 1 | 1 |
| Grade 2 | 3 | 0 | 2 | 2 |
| Grade 3 | 1 | 4 | 3 | 1 |
| Grade 4 | 0 | 0 | 1 | 0 |
| Not documented | 1 | 0 | 1 | 2 |
| **Dysphagia** |  |  |  |  |
| Grade 1 | 2 | 3 | 3 | 0 |
| Grade 2 | 2 | 3 | 4 | 0 |
| Grade 3 | 1 | 1 | 1 | 1 |
| Not documented | 1 | 0 | 1 | 5 |
| **Radiation-induced skin injury** |  |  |  |  |
| Grade 1 | 2 | 2 | 2 | 2 |
| Grade 2 | 4 | 5 | 4 | 2 |
| Grade 3 | 0 | 0 | 2 | 1 |
| Not documented | 0 | 0 | 1 | 1 |

ENI, elective nodal irradiation; CRT, chemoradiotherapy; SD, standard deviation

# Supplementary Table 3

Individual and summarized data for the planning target volume (PTV), whole-body dose (WBD), total and radiation-induced (RI) γH2AX/53BP1 foci per peripheral blood lymphocyte (PBL), proportions of total and radiation-damaged PBLs harboring at least one RI γH2AX/53BP1 focus per cell, and total and RI dicentric chromosomes.

**Supplementary Figure 3**

**Supplementary Figure 3:** Radiation-induced colocalizing γH2AX/53BP1 foci in peripheral blood lymphocytes post-chemoradiotherapy (CRT) for all patients stratified according to (A) definitive or adjuvant CRT or (B) elective nodal irradiation (ENI) status.

**Supplementary Figure 4**

**Supplementary Figure 4:** Radiation-induced γH2AX/53BP1 foci in patients’ peripheral blood lymphocytes (PBLs) after (A) definitive or (B) adjuvant radiotherapy. Data are shown relative to the planning target volume (left panel), whole-body dose (middle panel), and stratified by standard elective nodal irradiation (ENI) and delayed ENI (right panel) 15 min (upper panel) or 24 h (lower panel) after the first fraction. The lines in the left and middle panels represent linear fits to the data, along with the corresponding function, Pearson correlation coefficient (R), and the p-value of the fit. Statistical comparison between delayed and standard ENI (right panel) was conducted using Student's t-test (* p<0.05; ** p<0.01; *** p<0.001).

**Supplementary Figure 5**

**Supplementary Figure 5:** Analysis of the proportion of radiation-damaged peripheral blood lymphocytes (PBLs) and the distribution of biological damage indicators in patients. (A) Fraction of PBLs with at least one radiation-induced γH2AX/53BP1 focus after radiotherapy relative to planning target volume (left panel), whole-body dose (middle panel), and stratified by standard elective nodal irradiation (ENI) and delayed ENI (right panel) 24 h after the first fraction (upper panel) and before the sixth fraction (lower panel). The lines in the left and middle panels represent linear fits to the data, along with the corresponding function, Pearson correlation coefficient (R), and the p-value of the fit. (B) Distribution of radiation-induced γH2AX/53BP1 foci in patients’ PBLs 15 min (left panel) and 24 h after the first fraction (middle panel) and before the sixth fraction (right panel) of radiotherapy, stratified according to standard ENI and delayed ENI. Data are presented as the mean and standard deviation. Statistical comparison between radiotherapy ± ENI was conducted using Student's t-test (* p < 0.05; ** p < 0.01; *** p < 0.001).

# Supplementary Table 4

Timing and grade of radiation-induced lymphopenia (absolute lymphocyte counts <999/mm³) at nadir by radiotherapy technique.

| Onset of RIL (week after start of radiotherapy) | Standard ENI | Delayed ENI |
| --- | --- | --- |
| 1 | 38% (5/13) | 0% (0/12) |
| 2 | 23% (2/13) | 58% (7/12) |
| 3 | 31% (4/13) | 33% (4/12) |
| 4 | 8% (1/13) | 0% (0/12) |
| 5 | 0% (0/13) | 0% (0/12) |
| 6 | 0% (0/13) | 8% (1/12) |
|  |  |  |
| RIL Severity (CTCAE v5) |  |  |
| Grade I | 7% (1/14) | 0% (0/13) |
| Grade II | 21% (3/14) | 15% (2/13) |
| Grade III | 50% (7/14) | 77% (10/13) |
| Grade IV | 21% (3/14) | 8% (1/13) |

RIL, radiation-induced lymphopenia; EN, elective nodal; ENI, elective nodal irradiation; CTCAE v5, Common Terminology Criteria for Adverse Events Version 5.0.

# Supplementary Table 5

Individual and summarized data for absolute lymphocyte counts per µl collected pre-chemoradiotherapy (CRT) and subsequently every week during CRT and relative lymphocyte counts related to the pre-CRT baseline.

# Supplementary Table 6

## Key pathway gene set enrichment analysis results across primary contrasts.

| Pathway | DiD  Pre→Frac5 | DiD  Pre→End | Delayed ENI  Pre→End | Standard ENI  Pre→End |
| --- | --- | --- | --- | --- |
| Oxidative Phosphorylation | ↑ 2.71*** | ↑ 3.13*** | ↑ 3.14*** | ↓ -0.66 |
| Myc Targets V1 | ↑ 1.96*** | ↑ 2.47*** | ↑ 1.43* | ↓ -1.81*** |
| Interferon-α Response | ↑ 1.12 | ↑ 2.37*** | ↑ 2.84*** | ↑ 1.12 |
| Interferon-γ Response | ↓ -0.82 | ↑ 1.65** | ↑ 2.39*** | ↑ 1.65*** |
| Tnf-α Signaling via NF-κB | ↓ -1.51** | ↓ -0.84 | ↑ 1.7*** | ↑ 1.98*** |
| Inflammatory Response | ↓ -1.08 | ↓ -1.08 | ↑ 1.6** | ↑ 2.19*** |
| Mitotic Spindle | ↓ -1.23 | ↓ -2.61*** | ↓ -1.98*** | ↑ 1.64*** |
| Il6/Jak/Stat3 Signaling | ↓ -1.38 | ↓ -1.27 | ↑ 1.16 | ↑ 2.23*** |

ENI, elective nodal irradiation; ↑, enriched in elective nodal-sparing / increases in arm; ↓, enriched in standard ENI - / decreases in arm. *** FDR < 0.001, ** FDR < 0.01, * FDR < 0.05, no star, FDR ≥ 0.05; DiD, difference-in-differences; contrasts: positive = delayed ENI arm changes more in that direction.

**Supplementary Figure 6**

**Supplementary Figure 6:** Normalised expression counts of ISG15 (left) and PARP10 (right) across treatment arms and timepoints. Each point represents one sample; donor identity is encoded by shape and timepoint by colour (blue = pre-CRT, green = before sixth fraction, red = end-CRT).

**Supplementary Figure 7**

**Supplementary Figure 7:** Relative lymphocyte counts one week post-1^st^ fraction in the adjuvant cohort stratified according to chemotherapy status. Data are presented as the mean and standard deviation.
